# N-Acetylcysteine for the Treatment of Psychiatric Disorders: A Review of Current Evidence

**DOI:** 10.1155/2018/2469486

**Published:** 2018-10-22

**Authors:** Soo Liang Ooi, Ruth Green, Sok Cheon Pak

**Affiliations:** ^1^Centre for Complementary & Alternative Medicine, Singapore 247909, Singapore; ^2^School of Biomedical Sciences, Charles Sturt University, Bathurst, NSW 2795, Australia

## Abstract

N-acetylcysteine, a sulphur-containing amino acid for the treatment of paracetamol overdose and chronic obstructive pulmonary disease, is a widely available off-the-shelf oral antioxidant supplement in many countries. With the potential to modulate several neurological pathways, including glutamate dysregulation, oxidative stress, and inflammation that can be beneficial to the brain functions, N-acetylcysteine is being explored as an adjunctive therapy for many psychiatric conditions. This narrative review synthesises and presents the current evidence from systematic reviews, meta-analyses, and latest clinical trials on N-acetylcysteine for addiction and substance abuse, schizophrenia, obsessive-compulsive and related disorders, and mood disorders. Good evidence exists to support the use of N-acetylcysteine as an adjunct treatment to reduce the total and negative symptoms of schizophrenia. N-acetylcysteine also appears to be effective in reducing craving in substance use disorders, especially for the treatment of cocaine and cannabis use among young people, in addition to preventing relapse in already abstinent individuals. Effects of N-acetylcysteine on obsessive-compulsive and related disorders, as well as on mood disorders, remain unclear with mixed reviews, even though promising evidence does exist. Larger and better-designed studies are required to further investigate the clinical effectiveness of N-acetylcysteine in these areas. Oral N-acetylcysteine is safe and well tolerated without any considerable adverse effects. Current evidence supports its use as an adjunctive therapy clinically for psychiatric conditions, administered concomitantly with existing medications, with a recommended dosage between 2000 and 2400 mg/day.

## 1. Introduction

**Figure 1:**
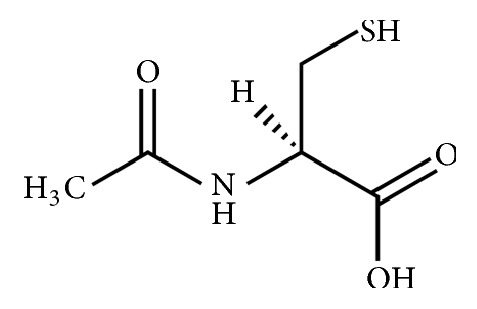
Structural formula of N-acetylcysteine.

N-acetylcysteine (molecular formula: C_5_H_9_NO_3_S) is an acetylated derivative of cysteine, a sulphur-containing amino acid (see Figure 1). As an antioxidant precursor to glutathione, N-acetylcysteine has been used as a prodrug in the clinical treatment of paracetamol overdose for over 30 years.[1] More recently, it has also been applied as a mucolytic in the treatment of chronic obstructive pulmonary disease, cystic fibrosis, and contrast-induced nephropathy.[1] N-acetylcysteine is widely available in many countries, including USA, Canada, and Australia, as an inexpensive off-the-shelf nutritional supplement commonly marketed as a potent antioxidant for brain functions. Increasingly, it is being explored as an adjunctive therapy for many psychiatric conditions. [1, 2] With early life stress found to be associated with the onset and the severity of many psychiatric conditions in adulthood, N-acetylcysteine is also a potential preventive therapy for young at-risk subjects. [3–5]

To facilitate evidence-informed decision-making by clinicians, researchers, and patients, this review synthesises findings from systematic reviews and meta-analyses of N-acetylcysteine for the following psychiatric conditions: addiction and substance abuse, schizophrenia, obsessive-compulsive and related disorders, and mood disorders. We conducted searches in research databases (EBSCOHost Psychology, PubMed, and ProQuest) to identify relevant studies published between 2007 and March 2018. We selected only English-language publications which included human clinical trials that studied the effects of N-acetylcysteine on one or more of the psychiatric conditions in our review. Based on the latest inclusion dates of the selected systematic reviews and meta-analyses, we also searched for newer randomised-controlled trials to augment the evidence base.

## 2. Mechanisms of Action

N-acetylcysteine is theorised to act via multiple pathways in the brain. Firstly, as the acetylated form of cysteine, it is bioavailable and able to cross the blood-brain barrier. Cysteine is the rate-limiting component in the production of the antioxidant glutathione. Several animal studies have shown evidence of increased brain glutathione following oral administration of N-acetylcysteine.[6, 7] Oxidative stress and reduced antioxidant status are common to many psychiatric disorders inclusive of schizophrenia, depression, bipolar, and obsessive-compulsive disorders.[1, 8] Increasing production of glutathione assists in the restoration of redox imbalance in these conditions.

Secondly, neurotransmitter dysregulation is evident throughout the spectrum of psychiatric conditions. N-acetylcysteine has shown promise for attenuating both dopamine and glutamate dysregulation. In one cell line study, N-acetylcysteine was found to significantly improve dopamine receptor binding and neuron survival.[9] Glutamate, in particular, is strongly implicated in the development and maintenance of conditions including obsessive-compulsive disorders and addiction.[10, 11] N-acetylcysteine regulates glutamate via the cysteine-glutamate antiporter (system Xc-) and glial glutamate transporter (GLT1), both essential components of glutamate homeostasis. System Xc- exchanges extracellular glutamate for intracellular cysteine on a 1:1 ratio, promoting the activation of mGlu2/3 receptors and inhibiting presynaptic release of glutamate.[12, 13] Reduced expression of the system Xc- and GLT1 is associated with higher levels of synaptic glutamate transmission, decreased tone on mGlu2/3 receptors, reinstatement of drug-seeking behaviour associated with addiction withdrawal, and the pathology of repetitive behaviours.[12, 13]

Thirdly, N-acetylcysteine may assist in the modulation of inflammatory pathways. Elevated levels of cytokines such as interleukin-6, C-reactive protein, and tumour necrosis factor alpha are evident in patients suffering from depression and other psychiatric disorders.[19–22] N-acetylcysteine may reduce inflammatory markers implicated in the development and maintenance of these conditions, influencing the inflammatory cascade directly as well as through amelioration of oxidative stress via redox reactions.[1, 23]

## 3. Clinical Evidence 

### 3.1. Addiction and Substance Use Disorders

Addiction and substance abuse are enormous issues worldwide, with few proven treatments available and limited success with behavioural therapies.[24] While research into substance abuse has traditionally focused on dopamine and associated reward-based behaviour, glutamate dysregulation has been suggested as another avenue related to the development and maintenance of addiction.[25] Preclinical results have shown N-acetylcysteine to be able to restore the imbalance of cysteine-glutamate exchange in the brain and decrease drug-seeking behaviours in animal models.[13] Indeed, treatment of addiction and substance abuse disorders with N-acetylcysteine has been an active area of research. Many clinical trials were conducted over the last decade with their results summarised in 5 systematic reviews (See Table 1) published within the last 4 years.

Asevedo et al. [14] performed the first systematic review of N-acetylcysteine for the treatment of addictions. This review included 9 clinical trials with a total of 295 participants. The authors suggested a potential role for N-acetylcysteine in the treatment of addiction, especially of cocaine and cannabis dependence, although methodological limitations exist for some randomised control trials, particularly in relation to small sample sizes.[14]

Deepmala et al. [15] systematically reviewed available evidence on the effectiveness of N-acetylcysteine in the treatment of psychiatric and neurological disorders. Based on the results of 19 included studies on addiction and substance use disorders, there is evidence for N-acetylcysteine in the treatment of cannabis and cocaine addiction, but results are inconsistent. Limited evidence was found for use of N-acetylcysteine in other types of addiction (methamphetamine, nicotine, and pathological gambling).[15]

A total of 18 clinical studies on substance use disorders was analysed by Minarini et al. [16], including 5 on cocaine use, 4 on cannabis use, 6 on nicotine addiction, 2 on methamphetamine use, and 1 on pathological gambling. This review found the available data to be preliminary in nature with no significant results on primary outcomes of most included studies. Positive evidence is mainly provided through analysis of secondary outcomes or analysis of subsamples.[16] The authors only found the clinical usefulness of N-acetylcysteine in the treatment of cannabis use disorder in young people.[16] Subsequently, a newer randomised placebo-controlled trial of N-acetylcysteine for cannabis use disorder published after the review also found no statistically significant evidence that the N-acetylcysteine and placebo groups differed in cannabis abstinence in adults.[10]

Nocito Echevarria et al. [17] systematically reviewed both animal studies and clinical trials available on N-acetylcysteine treatment for cocaine dependence. N-acetylcysteine was found to reduce craving, desire to use cocaine, cocaine-cue viewing-time and cocaine-related spending based on the findings from 4 clinical trials.[17] The positive effect was potentially due to the restoration of glutamate homeostasis as indicated in animal studies. Nevertheless, in one large double-blind placebo-controlled trial conducted with 111 cocaine-addicted treatment-seeking adults, N-acetylcysteine failed to affect abstinence.[30] Hence, the authors suggested that N-acetylcysteine might be better suited for relapse prevention in already abstinent individuals.[17]

The latest systematic review and meta-analysis by Duailibi et al. [18] found N-acetylcysteine to be significantly superior to placebo for reducing craving symptoms in substance abuse disorders. The result was derived from pooled analysis of 7 randomised control trials with heterogeneous methodology and a small sample size of 245.[18]

### 3.2. Schizophrenia

N-acetylcysteine acts at multiple points within the brain to potentiate activities that are beneficial to schizophrenia. These actions include modulating neuroinflammation associated with neuron dysfunction and apoptosis, promoting neurogenesis and repair of neuronal damage, as well as normalising glutamate dysregulation such as N-methyl-D-aspartate hypofunction.[1, 15, 31, 32] Patients treated with N-acetylcysteine showed increased multivariate phase synchronisation that altered the neuron connectivity of the brain, measured using electroencephalogram in one clinical trial, even before any clinically detectable improvement.[33]

Only 1 systematic review conducted exclusively for clinical trials of N-acetylcysteine in schizophrenia was found in the literature (See Table 2). Chen et al. [26] included only 2 double-blind, placebo-controlled trials and found adjunctive N-acetylcysteine may be efficacious in reducing negative and general symptoms of schizophrenia. A meta-analysis by Zheng et al. [27] that included 3 randomised control trials with 307 (N-acetylcysteine: 153, placebo:154) participants showed that N-acetylcysteine significantly improved total symptom scores in schizophrenia. Other related systematic reviews, including a Cochrane review on antioxidant treatment for schizophrenia, have also found N-acetylcysteine to be a promising add-on treatment for schizophrenia.[15, 34–37] The conclusions of these systematic reviews and meta-analyses were drawn mainly on the positive results from 2 double-blind, randomised, placebo-controlled trials.

A large multicentre clinical trial found taking 1000 mg of N-acetylcysteine twice daily to be more effective than taking a similar dose schedule of placebo in improving the total, negative, and general symptom scales of 140 chronic schizophrenia patients over a 24-week period.[38] N-acetylcysteine was used as an adjunctive treatment with the participants continued their maintenance antipsychotic medication throughout the study.[38]

Another smaller study examined a group of 46 patients in the active phase of schizophrenia, concurrently being treated with risperidone and N-acetylcysteine. Those treated with N-acetylcysteine (1000 mg/day in the first week increasing to 2000 mg/day for 7 weeks) achieved statistically significant improvements in the total and negative symptoms of schizophrenia compared to those on placebo, over the 8-week period.[39]

A large clinical trial is currently being conducted across 4 Australian sites to investigate the efficacy of N-acetylcysteine as an adjunctive medication to clozapine in the treatment of schizophrenia.[40] This multicentre, randomised, placebo-controlled trial aims to include 168 clozapine-resistant schizophrenia patients randomised to take 2000 mg/day of either N-acetylcysteine or placebo for 52 weeks. Positive results from this trial will certainly confirm N-acetylcysteine as an effective add-on treatment for schizophrenia.[40]

### 3.3. Obsessive-Compulsive and Related Disorders

Obsessive-compulsive disorder is a debilitating illness that can severely affect patients' quality of life. The development of this condition has long been associated with the dysfunction in the availability of serotonin transporter in the brain.[41] More recently, the role of the neurotransmitter glutamate has also been implicated in its pathogenesis. Patients with obsessive-compulsive disorder were found to have an increased level of glutamate in the cerebrospinal fluid.[41] N-acetylcysteine has been proposed as a novel treatment for this condition due to its ability to inhibit the synaptic glutamate release through the glial cysteine-glutamate exchange.[41] Other related disorders such as trichotillomania (hair pulling disorder), onychophagia (nail biting), Tourette syndrome, and excoriation (skin picking) also share some common neurobiology that can be potentially treated with N-acetylcysteine as a glutamate-modulating agent.[11]

A systemic review of N-acetylcysteine for the treatment of obsessive-compulsive and related disorders by Oliver et al. [11] (See Table 3) found encouraging results from 11 included studies (5 clinical trials and 6 case reports/series). Treatment with 2,400-3,000 mg/day of N-acetylcysteine in the included trials was found to reduce the severity of symptoms and demonstrate good tolerability with minimal adverse effects.[11] However, another systematic review by Smith et al. [28] which included only 4 methodologically robust clinical trials found the results on the treatment effects of N-acetylcysteine on obsessive-compulsive and related disorders remain inconclusive.

Minarini et al. [16] included 9 clinical trials on the treatment effects of N-acetylcysteine on obsessive-compulsive and related disorders (obsessive-compulsive disorder: 3, Tourette syndrome: 1, trichotillomania: 2, excoriation: 2, and onychophagia: 1) in their systematic review. Considering the results of these 9 clinical trials together with 11 published case reports/series, the authors found the findings remain preliminary. Among these conditions, excoriation appears to be the most promising area for N-acetylcysteine utilisation.[16]

Results from two newer clinical trials have become available after the publication of these systemic reviews. Ghanizadeh et al. [42] demonstrated N-acetylcysteine to be an effective add-on to citalopram in improving resistance/control to compulsions in children and adolescents with obsessive-compulsive disorder in a double-blind, placebo-controlled trial with 34 pediatric patients. Significant reduction in the score of resistance/control to obsessions was detected in the intervention group after supplementing with N-acetylcysteine (titrated up to 2400 mg/day) for 10 weeks. No significant change was observed in the placebo group.[42]

Costa et al. [43] did not find any significant benefit of N-acetylcysteine in reducing the severity of obsessive-compulsive symptoms among 40 treatment-resistant adults in a 16-week double-blind, placebo-controlled study of N-acetylcysteine (3,000 mg/day). The reduction of symptom scale measured in the N-acetylcysteine group was not significantly lower than the reduction in the placebo group. Nevertheless, N-acetylcysteine was found to be superior to placebo in reducing anxiety symptoms in secondary outcome analysis.[43]

### 3.4. Mood Disorders–Bipolar and Depression

Up until recently, treatments for mood disorders inclusive of major depression and bipolar were largely based on the pathology of the monoamine theory. Treatment options remain limited, and not all patients are responsive.[44] In addition to neurotransmitter dysregulation, research now links low mood to increased oxidative stress and dysfunction of glutamatergic systems, with N-acetylcysteine being explored as an adjunctive alongside primary antidepressant treatment. [45–47]

Fernandes et al. [29] (See Table 4) included 5 studies in a systematic review and meta-analysis of double-blind, placebo-controlled trials using N-acetylcysteine for depressive symptoms regardless of the main psychiatric condition. Pooled analysis with data from a total of 574 participants (N-acetylcysteine: 291, placebo: 283), N-acetylcysteine was shown to significantly ameliorate depressive symptoms and improve functionality compared to placebo.[29] The authors found insufficient data to reliably analyse the effects of N-acetylcysteine on quality of life and manic symptoms.[29]

Deepmala at al. [15] reviewed 8 studies (7 controlled and 1 uncontrolled) on the bipolar disorder and 2 studies (1 controlled and 1 uncontrolled) on the depressive disorder. N-acetylcysteine treatment was found to lessen symptoms of bipolar disorder but not affecting the frequency of cycling between mood states. Although the results for N-acetylcysteine treatment for depressive disorder remained mixed and further evidence was required, the authors accorded N-acetylcysteine to be a promising treatment option for mood disorders.[15]

Nine clinical trials (8 double-blind placebo-controlled trials plus 1 open-label trial) that studied the effects of N-acetylcysteine on bipolar patients, 1 double-blind placebo-controlled trials on N-acetylcysteine for major depression, and 1 case series on treatment-resistance major depression were reviewed by Minarini et al. [16]. Results from these studies were inconclusive. N-acetylcysteine was assessed to be a promising add-on treatment for depression in bipolar disorder only.[16]

Zheng et al. [27] meta-analysed 3 double-blind placebo-controlled trials of N-acetylcysteine on mood disorders (bipolar: 2, depressive disorder: 1). Their analysis found treatment with N-acetylcysteine had no significant effect on depressive and manic symptoms as assessed by the Young Mania Rating Scale in bipolar disorder and only a small effect on major depressive symptoms.[27]

A newer trial which explores effects of adjunctive N-acetylcysteine treatment on inflammatory and neurogenesis markers in the unipolar depression provided additional supporting evidence.[48] In this double-blind, placebo-controlled trials with 252 participants, N-acetylcysteine treatment of 2000 mg/day significantly improved depressive symptoms over the 16 weeks trial period compared to placebo.[48] However, the study failed to find any relevant inflammatory and neurogenesis marker that directly involved in the therapeutic mechanism of N-acetylcysteine in depression.[48] An upcoming 16-week trial which investigates N-acetylcysteine and a combination of other mitochondrial agents compared with placebo was described in the literature. The results from this trial are expected to be published soon.[49]

## 4. Safety and Adverse Effects

N-acetylcysteine has an excellent safety profile. An oral dose of N-acetylcysteine as high as 10x 2800 mg was evaluated for safety in a clinical study with no major adverse effect reported.[50] All major systematic reviews found N-acetylcysteine to be a well-tolerated oral therapy without any considerable adverse effects. [11, 15, 16, 18, 29]

Gastrointestinal symptoms including mild abdominal discomfort, heartburn, flatulence, cramps, nausea, vomiting, and diarrhoea were the most common adverse effects reported in clinical trials of N-acetylcysteine. [11, 15, 16, 18, 29] Other nonspecific side-effects reported were headaches, skin rashes, elevated blood pressure, dry mouth, fatigue, muscle pains, insomnia, nasal congestion, runny nose, restlessness, and dizziness. However, these are isolated incidents only with no consistent reporting of any severe incident due to N-acetylcysteine treatment. [11, 15, 16] In fact, N-acetylcysteine is observed to exert protective effects to adverse effects related to psychiatric medications when used as an adjunctive therapy.[39]

N-acetylcysteine can potentially interact with paracetamol, glutathione, and anticancer agents. It also strongly potentiates the effect of nitrates vasodilators and related medications, leading to the risk of hypotension.[16] As such, the use of N-acetylcysteine in patients taking these medications should be cautioned.

## 5. Discussion

There is plausible evidence suggesting N-acetylcysteine as a novel and effective treatment for certain psychiatric conditions based on current research. The bulk of the evidence exists for addictions and substance abuse disorders. N-acetylcysteine appears to be effective in reducing craving in the treatment of substance use disorders, notably for cocaine and cannabis use among young people, as well as preventing relapse in already abstinent individuals. However, with mixed results from different studies and reviews, more clinical trials are required to strengthen the evidence base. In contrast, good evidence exists to support the use of N-acetylcysteine as an adjunct treatment for schizophrenia based on a small number of well-designed trials. Positive results are shown consistently across these trials.

Although there is evidence of some benefits in applying N-acetylcysteine to augment the treatment of obsessive-compulsive and related disorders to reduce resistance/control to compulsions, overall results from clinical trials are mixed. Similarly, evidence on adjunctive N-acetylcysteine treatment for mood disorders, either bipolar or major depressive disorder, remains unclear with conflicting results found in different systematic reviews and clinical trials. Even though the supporting evidence is deemed promising, larger and better-designed studies are required to further investigate the clinical effectiveness of N-acetylcysteine in these areas. A point of interest to note is that many of the current research explored N-acetylcysteine as only an adjunctive treatment. To truly ascertain the therapeutic potential of N-acetylcysteine, clinical studies with N-acetylcysteine as a monotherapy are warranted.

The excellent safety profile of N-acetylcysteine coupled with the potential benefits demonstrated in many clinical trials and uncontrolled studies supports the continuing research effort in the study of N-acetylcysteine for the treatment of psychiatric disorders. The available evidence certainly supports N-acetylcysteine to be a viable adjunctive treatment option in clinical settings. Even though the therapeutic dose of N-acetylcysteine has yet to be ascertained, most studies were done with dosages between 2000 and 3600 mg/day. [14, 15, 17, 27] A dosage as high as 6000 mg/day has also been explored in clinical trials. Overall, a dose range between 2000 and 2400 mg/day was suggested to be effective and well tolerated.[15]

## 6. Conclusion

This article reviews the current evidence for the promising use of N-acetylcysteine in the treatment of psychiatric disorders. N-acetylcysteine shows potential to be able to modulate several neurological pathways, including glutamate dysregulation, oxidative stress and inflammation to bring about relief from the troubling symptoms of addictions and substance abuse disorders, schizophrenia, obsessive-compulsive and related disorders, and mood disorders.

Given that it has achieved an excellent safety profile and is readily accessible and inexpensive, N-acetylcysteine may well prove to be an exciting and novel treatment with which to tackle the mental health epidemic currently affecting the whole world. Further research is needed to ascertain the capability of N-acetylcysteine as a standard treatment for the conditions reviewed, both as an adjunctive as well as a monotherapy, in addition to its neuroprotective potential which may extend to other disorders of the brain.

## Figures and Tables

**Table 1:** Summary of included reviews: N-acetylcysteine for addiction and substance abuse disorders.

**Authors (Year)**	**Type**	**Inclusion**	**No. Studies (Study Size)**	**Conclusion**
Asevedo et al. (2014) [14]	SR	Clinical trials that assessed NAC with outcomes related to an addiction.	Total = 9 (n = 295): Cocaine = 3 (n = 60); Cannabis = 2 (n = 140); Nicotine = 2 (n = 51); Methamphetamine = 1 (n = 31); Gambling = 1 (n = 13)	Included studies suggest a potential role for NAC in the treatment of addiction, especially cocaine and cannabis dependence.

Deepmala et al. (2015) [15]	SR	Clinical trials of psychiatric and neurological disorders which reported a direct clinical effect of NAC as an outcome.	Total = 19 (n = 781): Cocaine = 5 (n = 168); Cannabis = 3 (n = 229); Nicotine = 6 (n = 253); Methamphetamine = 2 (n = 63); Gambling = 3 (n = 68)	Limited evidence for NAC as a treatment for addiction. Positive results for cocaine, but only for those who were abstinent. Some evidence for cannabis, even though results are inconsistent. Premature to make recommendations for or against the use of NAC in other types of addiction.

Minarini et al. (2017) [16]	SR	Clinical trials that assessed NAC use as the independent variable and clinical outcomes related to a psychiatric disorder.	Total = 18 (n = 711): Cocaine = 5 (n = 168); Cannabis = 4 (n = 252); Nicotine = 6 (n = 188); Methamphetamine = 2 (n = 63); Gambling = 1 (n = 40)	The clinical usefulness of NAC for SUDs, apart from cannabis use disorder in young people, is not currently supported by good enough evidence.

Nocito Echevarria et al. (2017) [17]	SR	Human or animal studies using NAC as an intervention for cocaine dependence.	Total (Cocaine) = 6 (n = 188) (Human trials only)	Promising data from preliminary studies, but results from a double-blind placebo trial was mainly negative. Current data suggest NAC may be better suited for avoiding relapse in already abstinent subjects.

Duailibi et al. (2017) [18]	SR+MA	RCTs of NAC for treatment of SUD with standardized assessment of craving.	Total = 7 (n = 245): Cocaine = 2 (n = 43); Cannabis = 1 (n = 89); Nicotine = 3 (n = 67); Methamphetamine = 1 (n = 46)	NAC was significantly superior for reducing craving symptoms compared to placebo (Hedges' g = 0.94; 95% CI: 0.55–1.33). NAC has a potential clinical use for craving in SUDs.

***Abbreviation***. Confidence interval (CI); meta-analysis (MA); N-acetylcysteine (NAC); randomised control trial (RCT); substance use disorder (SUD); systematic review (SR).

**Table 2:** Summary of included reviews: N-acetylcysteine for schizophrenia.

**Authors (Year)**	**Type**	**Inclusion**	**No. Studies (Study Size)**	**Conclusion**
Chen et al (2016) [26]	SR	Double-blind RCTs of NAC in schizophrenia.	Total = 2 (n = 186)	Adjunctive NAC may be effective in reducing negative and general symptoms in schizophrenia.

Zheng et al (2018) [27]	SR+MA	All published randomised RCTs of NAC for major mental disorders with meta-analysable data.	Total = 3 (n = 307)	N-acetylcysteine significantly improved total psychopathology in schizophrenia (SMD = -0.74, 95% CI: -1.43, -0.06; I^2^ = 84%, P = 0.03).

***Abbreviation***. Confidence interval (CI); meta-analysis (MA); N-acetylcysteine (NAC); randomised control trial (RCT); standard mean difference (SMD); systematic review (SR).

**Table 3:** Summary of included reviews: N-acetylcysteine for obsessive-compulsive and related disorders.

**Authors (Year)**	**Type**	**Inclusion**	**No. Studies (Study Size)**	**Conclusion**
Oliver el al. (2015) [11]	SR	Human clinical trials or case reports involving diagnosed OCD or related disorders in which NAC was prescribed.	Total = 11 (n = 206): OCD = 3 (n = 46); TTM = 4 (n = 94); Onychophagia = 2 (n = 28); Excoriation = 2 (n = 38)	Treatment with 2,400-3,000 mg/d of NAC in the included trials was found to reduce the severity of symptoms and demonstrate good tolerability with minimal adverse effects.

Smith et al. (2016) [28]	SR	Placebo RCTs investigated NAC for OCD and related disorders with behavioural outcome measures.	Total = 4 (n = 162): OCD = 1 (n = 48); TTM = 2 (n = 89); Onychophagia = 1 (n = 25)	Results remain inconclusive, but NAC may still be useful as a treatment for OCD and related disorders on an individual level given its safety records.

Minarini et al. (2017) [16]	SR	Clinical trials that assessed NAC use as the independent variable and clinical outcomes related to a psychiatric disorder.	Total = 20 (n = 421): OCD = 7 (n = 149); TS = 1 (n = 31); TTM = 4 (n = 92); Excoriation = 5 (n = 101); Onychophagia = 3 (n = 48)	Promising results were found in trials testing the use of NAC as an add-on treatment for excoriation. Preliminary evidence warrants further investigation of the possible effectiveness NAC for OCD and related disorders.

***Abbreviation***. Meta-analysis (MA); N-acetylcysteine (NAC); obsessive-compulsive disorder (OCD); randomised control trial (RCT); systematic review (SR); tourette syndrome (TS); trichotillomania (TTM).

**Table 4:** Summary of included reviews: N-acetylcysteine for mood disorders.

**Authors (Year)**	**Type**	**Inclusion**	**No. Studies (Study Size)**	**Conclusion**
Fernandes et al. (2016) [29]	SR + MA	Double-blind RCTs of NAC versus placebo in adult subjects with presence of depressive symptoms.	Total = 5 (n = 574): Bipolar = 2 (n = 224); MDD = 3 (n = 350)	Treatment with NAC improved depressive symptoms as assessed by MADRS and HDRS compared to placebo (SMD = 0.37; 95% CI = 0.19 to 0.55; P < .001).

Deepmala et al. (2015) [15]	SR	Clinical trials of psychiatric and neurological disorders which reported a direct clinical effect of NAC as an outcome.	Total = 10 (n = 793): Bipolar = 8 (n = 539); MDD = 2 (n = 254)	NAC may lessen symptoms of bipolar disorder but may not affect the frequency of cycling between mood states. Results of NAC treatment for MDD is still mixed with further evidence required.

Minarini et al. (2017) [16]	SR	Clinical trials that assessed NAC use as the independent variable and clinical outcomes related to a psychiatric disorder.	Total = 12 (n = 868): Bipolar = 10 (n = 614); MDD = 2 (n = 254)	Results remain inconclusive with potential clinical application of NAC for depressive symptoms in bipolar disorder.

Zheng et al. (2018) [27]	SR+MA	All published randomised RCTs of NAC for major mental disorders with meta-analysable data.	Total = 3 (n = 394): Bipolar = 2 (n = 125); MDD = 1 (n = 269)	NAC had no significant effect on depressive and manic symptoms as assessed by the YMRS in bipolar disorder and only a small effect on major depressive symptoms.

***Abbreviation***. Confidence interval (CI); standard mean difference (SMD); Hamilton Depression Rating Scale (HDRS); major depressive disorder (MDD); meta-analysis (MA); Montgomery-Asberg Depression Rating Scale (MADRS); N-acetylcysteine (NAC); randomised control trial (RCT); systematic review (SR); Young Mania Rating Scale (YMRS).
